# The Fungal Iron Chelator Desferricoprogen Inhibits Atherosclerotic Plaque Formation

**DOI:** 10.3390/ijms21134746

**Published:** 2020-07-03

**Authors:** László Potor, Katalin Éva Sikura, Hajnalka Hegedűs, Dávid Pethő, Zsuzsa Szabó, Zsuzsa M Szigeti, István Pócsi, György Trencsényi, Dezső Szikra, Ildikó Garai, Tamás Gáll, Zsolt Combi, János Kappelmayer, György Balla, József Balla

**Affiliations:** 1 HAS-UD Vascular Biology and Myocardial Pathophysiology Research Group, Hungarian Academy of Sciences, University of Debrecen, 4012 Debrecen, Hungary; placi85@gmail.com (L.P.); loreleiy77@gmail.com (K.É.S.); gall.tamas@med.unideb.hu (T.G.); 2 Department of Pediatrics, Faculty of Medicine, University of Debrecen, 4012 Debrecen, Hungary; 3 Division of Nephrology, Department of Medicine, Faculty of Medicine, University of Debrecen, 4012 Debrecen, Hungary; hegedushajnalkababa@gmail.com (H.H.); davenicessus@gmail.com (D.P.); combizsolt@gmail.com (Z.C.); 4 Department of Molecular Biotechnology and Microbiology, Institute of Technology, Faculty of Science and Technology, University of Debrecen, 4012 Debrecen, Hungary; szabo.zsuzsa@science.unideb.hu (Z.S.); szigeti.zsuzsa@science.unideb.hu (Z.M.S.); pocsi.istvan@science.unideb.hu (I.P.); 5 Scanomed Ltd., University of Debrecen, 4012 Debrecen, Hungary; trencsenyi.gyorgy@med.unideb.hu (G.T.); szikra.dezso@med.unideb.hu (D.S.); garai@internal.med.unideb.hu (I.G.); 6 Department of Laboratory Medicine, Faculty of Medicine, University of Debrecen, 4012 Debrecen, Hungary; kappelmayer@med.unideb.hu

**Keywords:** atherosclerosis, lipid peroxidation, inflammation, siderophore, apolipoprotein e knockout mouse, positron emission tomography

## Abstract

Hemoglobin, heme and iron are implicated in the progression of atherosclerosis. Therefore, we investigated whether the hydrophobic fungal iron chelator siderophore, desferricoprogen (DFC) inhibits atherosclerosis. DFC reduced atherosclerotic plaque formation in ApoE^−/−^ mice on an atherogenic diet. It lowered the plasma level of oxidized LDL (oxLDL) and inhibited lipid peroxidation in aortic roots. The elevated collagen/elastin content and enhanced expression of adhesion molecule VCAM-1 were decreased. DFC diminished oxidation of Low-density Lipoprotein (LDL) and plaque lipids catalyzed by heme or hemoglobin. Formation of foam cells, uptake of oxLDL by macrophages, upregulation of CD36 and increased expression of TNF-α were reduced by DFC in macrophages. TNF-triggered endothelial cell activation (vascular cell adhesion molecule-1 (VCAM-1), intercellular adhesion molecules (ICAMs), E-selectin) and increased adhesion of monocytes to endothelium were attenuated. The increased endothelial permeability and intracellular gap formation provoked by TNF-α was also prevented by DFC. DFC acted as a cytoprotectant in endothelial cells and macrophages challenged with a lethal dose of oxLDL and lowered the expression of stress-responsive heme oxygenase-1 as sublethal dose was employed. Saturation of desferrisiderophore with iron led to the loss of the beneficial effects. We demonstrated that DFC accumulated within the atheromas of the aorta in ApoE^−/−^ mice. DFC represents a novel therapeutic approach to control the progression of atherosclerosis.

## 1. Introduction

The pathomechanism of the atherosclerotic vascular disorders, which is the leading cause of death worldwide, is based on two main distinct concepts, the lipid and the inflammation theories. Besides, lipids modification and oxidation are the initial steps of atherosclerosis, which provoke inflammation at the site of the artery wall [1]. There is a third approach, the lipid-inflammation synergy hypothesis since their signs can be observed parallel in atheromas [2]. Although the pathological deviation of plasma lipid profile is required to develop lipid plaques in most of the atherosclerotic cases, the oxidative modification of the Low-density Lipoprotein (LDL) is also a necessary step to generate foam cells in the artery wall [3,4]. Previous studies suggest that smooth muscle cells contribute at least 50% of the origin of foam cells as compared to macrophages in human and mouse atheromas. Moreover, the expression of smooth muscle cells markers, including smooth muscle cell α-actin and α-tropomyosin were decreased in smooth muscle cells during foam cell formation, while smooth muscle cells have become positive for macrophage markers, as well as CD68 and Mac-2 and transdifferentiated into endocytic phenotype [5,6].

LDL oxidation is initiated by reactive oxygen species (ROS) and catalyzed by transition metals localized to the arterial subendothelial space and can also be observed both in human and animal plasmas [7,8,9]. ROS are primarily derived from inflammatory cells, but resident cells of the vessel wall can also produce free radicals in a pathological situation. Transition metals, particularly iron, the most common transition metal in the human organism, and copper, are present in the arteriosclerotic vessels [10]. Forty years ago, in 1981, Jerome Sullivan first raised the “iron hypothesis” which linked iron to cardiovascular disease [11]. Steinberg described the importance of oxidative modification of LDL as the essential alteration of the lipoprotein to form foam cells through the particle uptake by macrophage scavenger receptors [12,13]. Esterbauer was able to catalyze LDL oxidation by copper [14], but these days the focus is on iron since its presence always can be demonstrated in severe atherosclerotic lesions.

Hard evidence shows that inflammatory hyperactivity, together with increased expressions of adhesion molecules such as vascular cell adhesion molecule-1 (VCAM-1), intercellular adhesion molecules (ICAMs) and CD62 antigen-like family member E (E-selectin) on vascular endothelial cells are the hallmarks of early atherosclerosis by recruiting inflammatory cells to the vessel wall facilitating their transendothelial migration [15]. Importantly, the expression of adhesion molecules is strongly induced by oxidized LDL (oxLDL) [12]. Uptake of oxLDL by smooth muscle cells and monocytes results in transdifferentiation into foam cells [6,16]. This transformation is regarded as a progressive step of the atherosclerotic marching of the vessel wall by the production of a wide variety of proinflammatory cytokines leading to lipid-inflammatory synergy [17,18,19,20]. The existence of this synergism is underlined by the beneficial effect of some anti-inflammatory agents, for example targeting interleukin-1 beta pathway, or antimalarial medicines, chloroquine, hydroxychloroquine as immunomodulatory drugs [21]. Interestingly, other anti-inflammatory agents, such as losmapimod [22] or methotrexate, fail to reduce the risk for cardiovascular events [23]. Numerous studies demonstrated that steroid hormones with anti-inflammatory properties enhance hypertension, ventricular remodeling, myocardial ischemia and atherothrombotic events in humans [24]. Furthermore, synthetic inhibitors of Phospholipase A_2_ failed to exhibit benefits in inflammatory cardiovascular diseases [25]. Unfortunately, there are no clinical trials that give evidence of a beneficial effect of anti-inflammatory drugs in atherosclerosis [26,27].

Iron is the most abundant transition metal in mammals, the total amount of iron in the human body is about 3.5 g (50 mg/kg) [28]. The iron in the human body is distributed mostly within hemoglobin (Hb, 65%), in myoglobin (4%), stored in ferritin and hemosiderin (15 to 30%) and bound in the plasma transferrin (0.1%) [29,30]. Unbound, labile form of reduced iron (Fe^2+^) catalyzes hydroxyl radical formation through the Fenton reaction, thus promotes the generation of ROS [29,31]. Numerous studies demonstrate that lipid peroxidation mediated by excess nonprotein-bound iron is potentially toxic to cells and tissues, subsequently may increase the risk for atherosclerosis [32,33]. Contrary to that, others have shown that iron overload attenuates the progression of atherosclerosis in animal models [34,35].

The solubility properties of the different iron complexes are taken into account in several experimental conditions. The hydrophobic nature of an iron complex is not always beneficial. If the complex allows iron to participate in free radical reactions, it may exacerbate injuries due to the incorporation of iron into the hydrophobic domains of cells [36]. Heme is the most abundant natural hydrophobic iron complex of the human body. We have previously shown that free heme liberated from hemoproteins is extremely cytotoxic sensitizing endothelial cells towards free radicals [37] and catalyzes LDL oxidation [3]. Since the heme hypothesis in vascular diseases has been raised by our group, it is extremely intriguing for us to study the nature of the two hydrophobic iron complex, desferricoprogen (DFC) and heme in an animal model and in vitro cell culture, which may allow us to draw therapeutic conclusions.

It has been shown that intraplaque hemorrhages frequently occur in atheromatous plaques due to the rupture of neovascularized vessels [38]. Oxidative plaque materials such as oxLDL and oxidized plaque lipids (oxPL) promote lysis of red blood cells (RBCs) and consecutive oxidation of liberated Hb at the site of atheromas. Oxidized Hb can release its heme group [39] resulting in intensified lipid oxidation in atheromatous plaques [40,41]. There is evidence that RBCs promote oxidation in early-stage atheroma in humans [42].

Siderophores are small organic compounds that are produced by bacteria, fungi, or plants and have a property to acquire iron with high-affinity from their environment [43,44]. Desferrioxamine B (DFO) abrogates tissue damage via preventing the oxidative processes and subsequently inflammation [45,46] and inhibits TNFα-induced expression of adhesion molecules [47]. Furthermore, DFO reduces atherosclerotic lesion development via inhibiting inflammation in Apolipoprotein-E-deficient (ApoE^−/−^) mice [48]. In contrast, others have shown that DFO increases mRNA expressions of inflammatory and proinflammatory genes in human intestinal epithelial cells [49]. These observations may represent indirect cellular effects since DFO is a classical hydrophilic compound, its permeability through cell membranes is limited [50,51]. One may speculate that DFO chelates the extracellular, hydrophilic iron pool with secondary cellular consequences.

**Figure 1:**
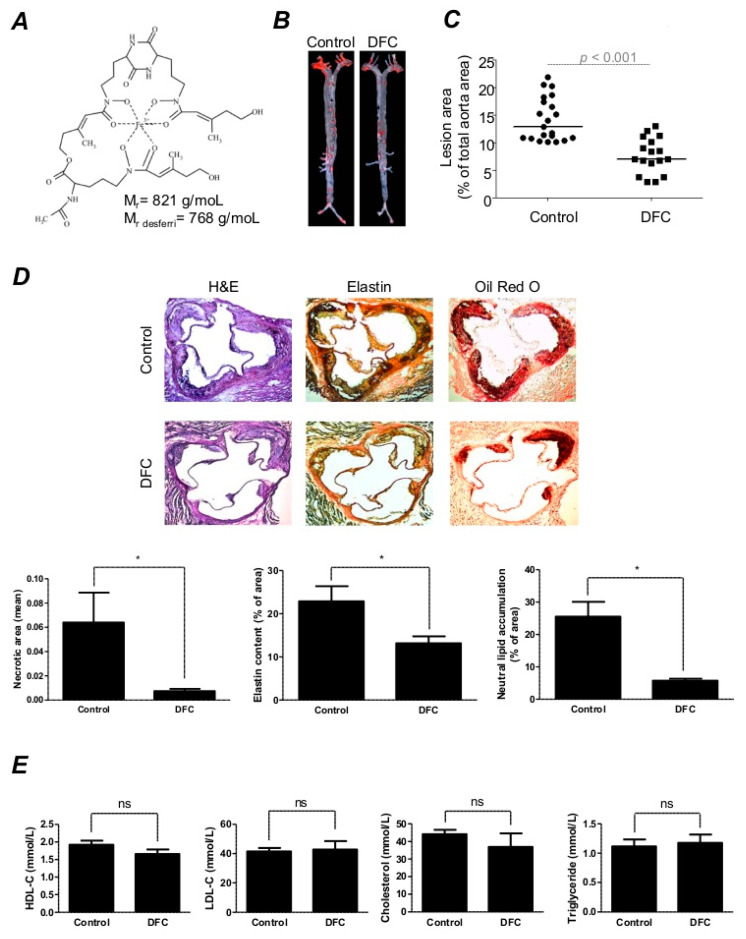
Intraperitoneal administration of desferricoprogen (DFC) attenuates atherosclerosis in mice. Eight-week-old Apolipoprotein-E-deficient (ApoE^−/−^) mice were fed ad libitum with an atherogenic diet containing 5% fat and 1.25% cholesterol and divided into DFC group (*n* = 17) receiving 160 mg/kg intraperitoneal DFC and control group (*n* = 21) receiving physiological saline every second day for 8 weeks. (**A**) Molecular structure of the coprogen. (**B**) Atherosclerotic lesions were examined by Oil Red O staining of en face aortas derived from DFC-treated or control mice. Scale bar: 1 mm. (**C**) Quantitative analysis of atherosclerotic plaque burden in Oil Red O-stained aortas using Image J software. (**D**) Immunohistochemical analysis of aortic cryosections (6 µm) derived from control and DFC-treated mice stained for Hematoxylin-Eosin (first row), Elastin (second row) and Oil Red O (third row) was performed with Image J software. Scale bar: 0.2 mm. (**E**) Quantitative analysis of plasma high-density lipoprotein-cholesterol (HDL-C), low-density lipoprotein-cholesterol (LDL-C), cholesterol and triglycerides levels of control and DFC-treated mice on an atherogenic diet. The graph shows the mean ± SEM of 6 mice plasma cholesterol levels per group. *p < 0.05; ns: not significant.

The goal of the present study is to provide data for the lipid and iron interaction in atherosclerosis to strengthen the importance of the two natural iron complexes, DFC and heme, in plaque development. We test whether the hydrophobic fungal siderophore DFC (Figure 1A) [52], produced by *Neurospora crassa*, owns beneficial effects in a widely used animal model for atherosclerosis that is ApoE^−/−^ mice. Additionally, we characterize the effect of DFC in a cell-specific manner focusing on inflammatory signs, such as the expression of adhesion molecules and gap formation in endothelial cells in response to TNF-α. Using monocyte adhesion, and foam cell formation assays we intend to gain information on whether DFC has a beneficial effect on cellular functions triggered by heme oxidized LDL.

## 2. Results

### 2.1. DFC Attenuates High Fat Diet-Induced Atherosclerotic Plaque Formation in ApoE^−/−^ Mice

To investigate whether DFC has an antiatherogenic effect, we employed an atherosclerotic animal model of ApoE deficiency. In ApoE^−/−^ mice fed with an atherogenic diet, an extensive plaque formation appeared en face of the aorta after eight weeks (Figure 1B, left panel). As shown in Figure 1B (right panel), intraperitoneal injection of DFC inhibited plaque formation compared to the physiological saline-injected control. Quantification of the atherosclerotic lesion in the entire aorta of the mice showed a significantly reduced area of plaque in the DFC group (*n* = 17) as compared to the control group (*n* = 21, Figure 1C). Immunohistochemistry stainings (H&E, Elastin and Oil Red O) of aortic roots confirmed the beneficial effect of DFC treatment (Figure 1D). DFC lowered the accumulation of lipids and decreased the deposition of elastin. As previously described in ApoE^−/−^ mice, the plasma high-density lipoprotein (HDL) cholesterol level decreased while the LDL cholesterol level increased leading to the progression of atherosclerosis [53]. Therefore, we assessed whether DFC treatment alters the plasma cholesterol concentration in mice. As shown in Figure 1E, there were no significant differences between the HDL cholesterol, LDL cholesterol, cholesterol and triglycerides levels in DFC-treated animals as compared to the control group (Figure 1E).

### 2.2. DFC Inhibits Lipid Peroxidation of Plaque Lipids and LDL in ApoE^−/−^ Mice as well as Heme/Hemoglobin-Catalyzed Oxidation of Lipid Derived from Human Carotid Artery Plaque and LDL

**Figure 2:**
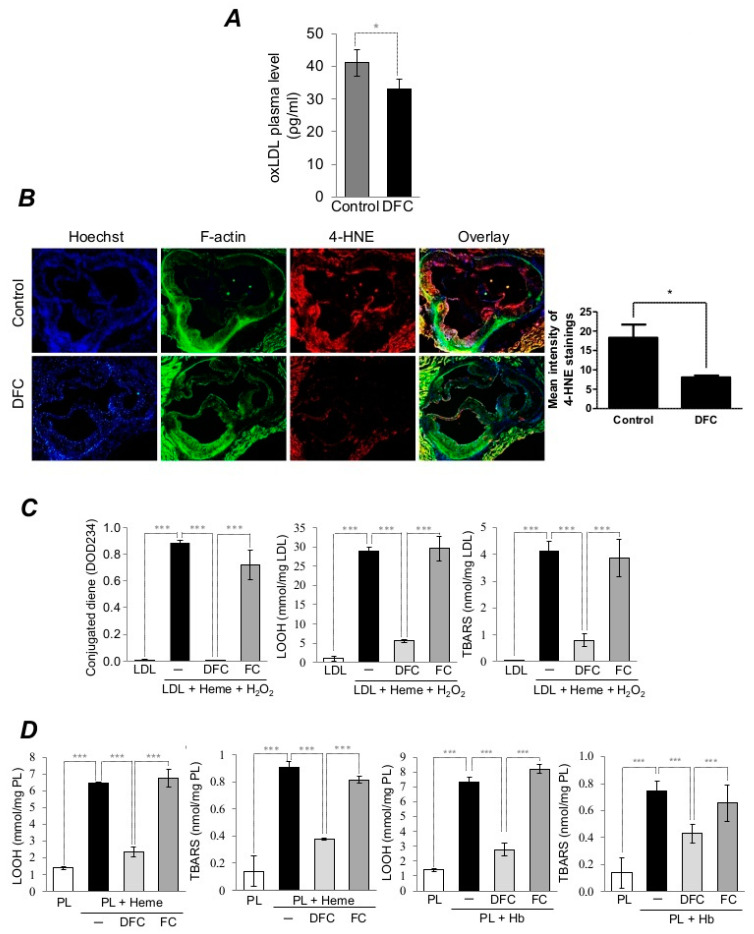
DFC inhibits lipid peroxidation of plaque lipids and LDL in ApoE^−/−^ mice and suppresses heme/hemoglobin-catalyzed oxidation of lipid of human atheromatous plaque and LDL. Eight-week-old ApoE^−/−^ mice were fed ad libitum with an atherogenic diet containing 5% fat and 1.25% cholesterol and divided into DFC group receiving 160 mg/kg intraperitoneal DFC and control group receiving physiological saline every second day for 8 weeks. (**A**) Quantitative analysis of plasma oxidized LDL (oxLDL) levels of DFC-treated and control mice (*n* = 5) are shown. (**B**) Immunofluorescent analysis of lipid peroxidation in the aortas of DFC-treated and control mice. Aortas (5 per group) were stained for DNA (Hoechst 33258, blue), F-actin (cytoskeleton, phalloidin-FITC, green) and 4-Hydroxynonenal (4-HNE, AF647, red). Quantification of 4-HNE mean intensity was performed with Image J software. Scale bar: 0.2 mm. (**C**) Conjugated dienes, lipid hydroperoxides (LOOH) and thiobarbituric-acid-reactive substances (TBARs) contents of LDL samples exposed to heme (5 µmol/L) and H_2_O_2_ (75 µmol/L) in the presence or absence of DFC (50 µmol/L) or FC (50 µmol/L) were measured. (**D**) LOOH and TBARs contents of plaque lipids (1 mg/mL) exposed to heme (5 µmol/L) or Hb (5 µmol/L) in the presence or absence of DFC (50 µmol/L) or FC (50 µmol/L) were measured. The graph shows the mean ± SEM of three separate experiments. *p < 0.05; ***p < 0.001.

Oxidative stress and lipid peroxidation are implicated in the pathogenesis of atherosclerosis [54]. To confirm that lipid peroxidation was inhibited by DFC, we measured the level of oxLDL in plasma derived from DFC-treated mice and control animals. The concentrations of oxLDL in DFC-treated mice were significantly lower than in the control mice (Figure 2A). Furthermore, we examined the extent of oxidative injury by performing immunofluorescence staining for the cytotoxic lipid peroxidation product 4-Hydroxynonenal (4-HNE) from the aortic root in control and DFC-treated mice. Strong 4-HNE staining was observed in the aorta derived from control mice whereas 4-HNE level was markedly lower in aorta derived from DFC-treated mice (Figure 2B). To further confirm that DFC inhibits lipid peroxidation catalyzed by heme-iron or Hb, we employed in vitro experiments. LDL and lipids derived from atheroma of carotid artery plaque (PL) were exposed to heme or Hb in the presence or absence of DFC or iron-saturated DFC (FC). The process of the oxidation was monitored by the measurement of three lipid peroxidation markers such as conjugated diene, lipid hydroperoxides (LOOH) and thiobarbituric-acid-reactive substances (TBARS). Incubation of LDL with heme or PL with heme or Hb led to lipid peroxidation, while DFC inhibited such oxidation. FC failed to prevent the oxidation of lipids (Figure 2C,D).

### 2.3. DFC Prevents TNF-α-Induced Expressions of Adhesion Molecules as well as Endothelial Cell Activation

**Figure 3:**
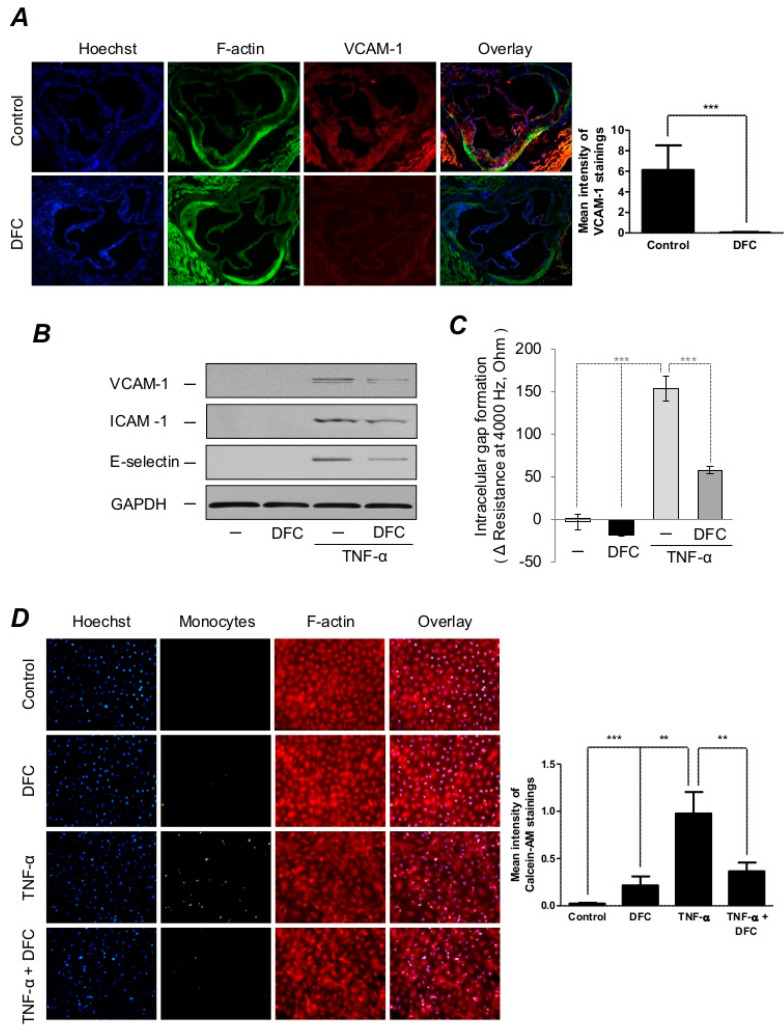
DFC prevents TNF-α-induced expression of adhesion molecules in ApoE^−/−^ mice and endothelial cells, preserves endothelial integrity and inhibits monocyte adhesion. (**A**) 8-week-old ApoE^−/−^ mice were fed ad libitum with an atherogenic diet containing 5% fat and 1.25% cholesterol and divided into DFC group (*n* = 17) receiving 160 mg/kg intraperitoneal DFC and control group (*n* = 21) receiving physiological saline every second day for 8 weeks. Aortas (5 per group) were stained for DNA (Hoechst 33258, blue), F-actin (cytoskeleton, phalloidin-FITC, green) and VCAM-1 (AF647, red), respectively. Quantification of VCAM-1 mean intensity was performed with Image J software. Scale bar: 0.2 mm. (**B**) Human Umbilical Vein Endothelial Cells (HUVEC) were incubated with or without DFC (50 µmol/L) for 16 h and treated with or without 1 ng/mL of TNF-α for 8 h. Representative Western blots showing VCAM-1, ICAM-1 and E-selectin protein expression in HUVEC normalized to GAPDH are shown. (**C**) HUVEC were cultured with or without DFC (50 µmol/L) for 16 h in CM199 medium containing 5% FBS. Then, cells were incubated in the presence or absence of TNF-α (1 ng/mL) for 3 h. Transendothelial electrical resistance was monitored by the Electric Cell-substrate Impedance Sensing (ECIS) Zθ instrument over 3 h. (**D**) Confluent HUVEC were incubated with or without DFC (50 µmol/L) for 16 h in CM199 medium containing 5% FBS. Then, cells were incubated with or without TNF-α (1 ng/mL) for 6 h followed by coincubation with calcein-labeled (5 µmol/L) monocytes for 30 min at 37 °C. Cells were then fixed and stained for DNA (Hoechst 33258, blue) and F-actin (cytoskeleton, red, iFlour 647). Quantification of Calcein-AM mean intensity was performed with Image J software. Images were taken with a fluorescent microscope at a magnification of 400×. ** *p* < 0.01; *** *p* < 0.001.

Atherosclerosis is an inflammatory disease accompanied by the expression of adhesion molecules, cytokines, chemokines and other mediators of inflammation. These molecules trigger oxidative stress and cell activation, respectively [15]. Therefore, we investigated whether DFC could attenuate the expression of vascular cell adhesion molecule-1 (VCAM-1) in ApoE^−/−^ mice. Immunofluorescence staining showed robust VCAM-1-positive staining in ApoE^−/−^ mice fed with an atherogenic diet, whereas we observed markedly lower staining in aorta derived from ApoE-deficient mice on an atherogenic diet treated with DFC (Figure 3A). TNF-triggers endothelial cell activation characterized by elevated expression of adhesion molecules such as VCAM-1, ICAM-1, E-selectin and increased intercellular gap formation [55,56]. Consequently, we examined the potential of DFC to control endothelial responses via inhibiting the expression of the TNF-α-induced adhesion molecules in endothelial cells. We found that DFC+TNF-α-treated endothelial cells expressed significantly lower levels of adhesion molecules compared to the TNF-α-treated cells (Figure 3B.). The recruitment of monocytes to the endothelium is an initial stage of atherosclerosis [57]. Therefore, we examined whether DFC attenuates the TNF-α-induced monocyte adhesion to the endothelium in vitro. We found that TNF-α markedly promoted monocyte adhesion to endothelium, whereas DFC pretreatment inhibited monocyte-endothelial cell interaction (Figure 3D). Next, we studied whether DFC prevents the perturbation of endothelial monolayer integrity triggered by TNF-α using the Electric Cell-substrate Impedance Sensing (ECIS) method. We showed that TNF-α treatment increased intracellular gap formation in the endothelial cell monolayer, while DFC pretreatment improved the monolayer integrity of endothelial cells against TNF-α-induced gap formation (Figure 3C).

### 2.4. DFC Attenuates LDL-Induced Oxidative Stress Catalyzed by Heme

**Figure 4:**
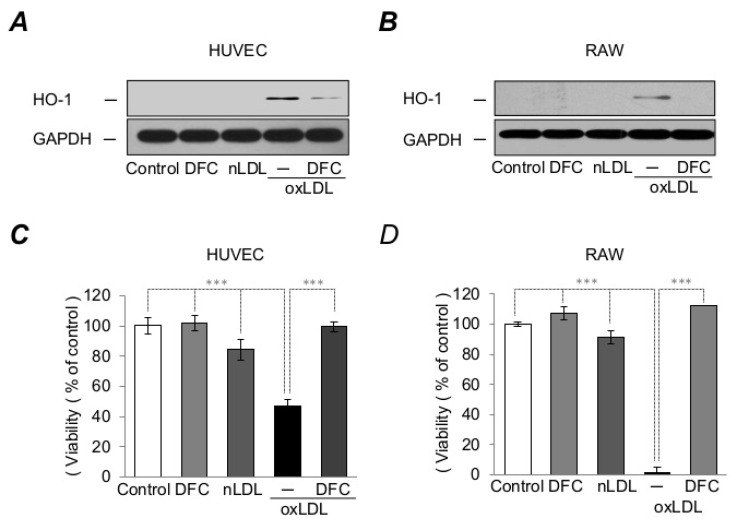
DFC inhibits LDL oxidation provoked endothelial cytotoxicity and oxidative stress catalyzed by heme. HUVEC and RAW 264.7 cells were cultured in the presence or absence of DFC (50 µmol/L) for 16 h. Next, cells were treated with native LDL (75 µg/mL) or oxLDL (75 µg/mL) in Hank’s Balanced Salt Solution (HBSS+) for 6 h for cell viability analysis or 8 h for Western blot analyzes. (**A**,**B**) Representative Western blots showing HO-1 protein expression in HUVEC and RAW 264.7 cells normalized to GAPDH are shown. (**C**) Cell viability of HUVEC and (**D**) RAW 264.7 cells were assessed by MTT assay. The graph shows the mean ± SEM of three separate experiments. *** *p* < 0.001.

We have shown that oxLDL triggers cellular heme oxygenase-1 (HO-1) expression, an enzyme protecting against diverse cellular stresses [58]. HO-1 also represents an excellent indicator of oxidizing stimuli. In our previous work, we have observed that DFC effectively inhibits oxLDL-induced oxidative stress and cytotoxicity in endothelial cells [52]. This prompted us to examine whether DFC attenuates oxLDL-induced oxidative damage that was followed up by the analysis of HO-1 expression. We showed that sublethal concentration of oxLDL (50 µg/mL) provoked the expression of the stress-responsive protein HO-1 in endothelial cells as well as in macrophages which were abrogated by DFC (50 µmol/L) in both cell types (Figure 4A,B). Subsequently, we aimed to verify our earlier results whether cell death provoked by oxLDL could be prevented by DFC in endothelial cells and macrophages at the toxic dose of 200 µg/mL. Our results showed that oxLDL reduced the viability of both endothelial cells (by 62%) and macrophages (by 97%) that were markedly reduced by DFC (50 µmol/L, Figure 4C,D). These results suggest that DFC effectively attenuates oxidative stress and cytotoxicity in resident cells of the vessel wall.

### 2.5. DFC Inhibits Foam Cell Formation and Suppresses Macrophage Activation Resulted from Heme-Catalyzed LDL Oxidation

**Figure 5:**
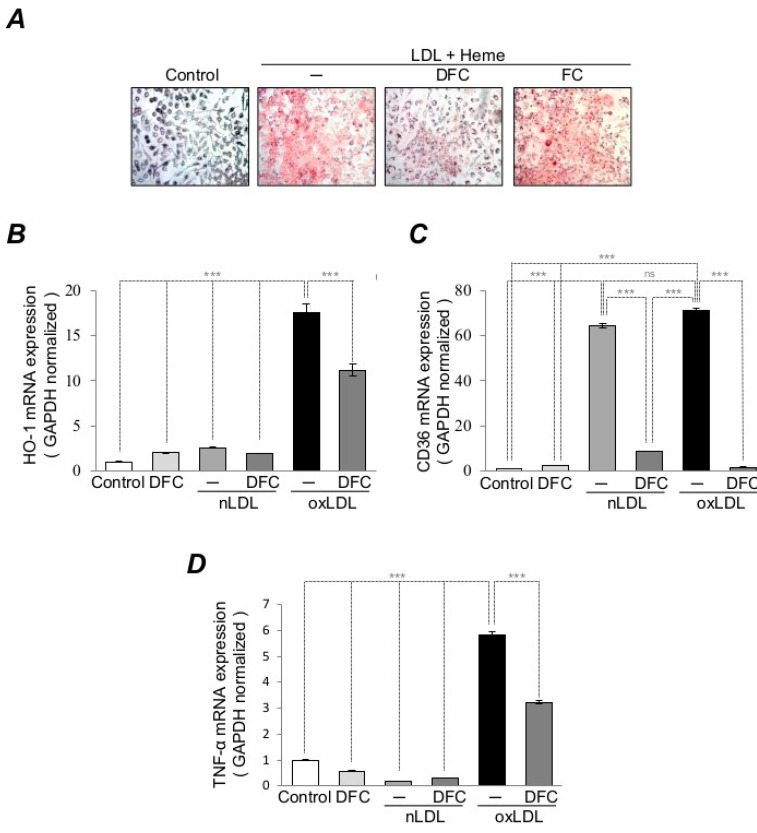
DFC inhibits foam cell formation and suppresses macrophage activation provoked by heme-catalyzed LDL oxidation. (**A**) RAW 246.7 macrophages were preincubated with DFC (50 µmol/L) or FC (50 µmol/L) for 16 h, then exposed to LDL (100 µg/L)-heme (5 µmol/L) solution in the presence or absence DFC (50 µmol/L) and FC for (50 µmol/L) for 24 h. Representative Oil Red O staining of RAW 246.7 macrophages are shown. (**B**–**D**) RAW 264.7 cells were grown in 6-well plates and pretreated with DFC (50 µmol/L) for 16 h. Cells were then exposed to native LDL (50 µg/mL) or oxLDL (50 µg/mL) for 6 h. Relative mRNA expression for HO-1, CD36 and TNF-α was assessed by Real-Time qPCR The graph shows the mean ± SEM of three separate experiments. ***p < 0.001, ns: not significant.

LDL oxidation and inflammatory cell accumulation are linked to the pathological changes of the vessel wall. Transdifferentiation of macrophages into foam cells in response to oxLDL and inflammation are the hallmarks of early atherosclerosis [19,59,60]. Based on the beneficial effects of DFC on LDL oxidation, we next examined whether DFC inhibits macrophage foam cell formation in response to oxLDL. Macrophages treated with oxLDL (50 µg/mL) showed a significant increase in foam cell formation as compared to the control native LDL (nLDL) group, while foam cell formation was blunted when macrophages were pretreated with DFC (100 µmol/L) demonstrated by the presence of less Oil red O droplets in cells of the DFC group (Figure 5A). The sublethal concentration of oxLDL represents oxidative stress for macrophages, and HO-1 mRNA expression can be used as a valuable tool to quantify the level of this injury. RAW 246.7 macrophages were pretreated with DFC (50 µmol/L) for 16 h to test whether DFC had inhibitory potential against oxLDL-induced stress reaction. A 6 h stress induced by oxLDL (50 µg/mL) resulted in a 17-fold increase of HO-1 mRNA expression, nevertheless pretreatment of cells with DFC inhibited significantly the HO-1 mRNA induction (Figure 5B). CD36, a scavenger receptor that imports fatty acids such as oxLDL into cells, is implicated in atherogenesis [61]. Moreover, components of oxLDL upregulate CD36 expression in macrophages [62]. To investigate whether DFC inhibits oxLDL-mediated upregulation of CD36 expression, we pretreated RAW cells with DFC (50 µmol/L) for 16 h, then, cells were treated with nLDL (50 µg/mL) or oxLDL (50 µg/mL) for 6 h. As shown (Figure 5C), both oxLDL and nLDL induced CD36 expression in macrophages which were significantly prevented by DFC pretreatment. In macrophages, oxLDL has been shown to provoke the massive release of TNF-α [63], a potent proinflammatory cytokine that is involved in the progression of atherosclerosis [64]. In contrast to CD36 expression, here, we showed that oxLDL but not nLDL triggered a massive TNF-α expression in macrophages which was ameliorated by DFC pretreatment (Figure 5D). These results suggest that DFC is a potent inhibitor of LDL oxidation and CD36 as well as TNF-α production in response to oxLDL in macrophages.

### 2.6. DFC Accumulates within the Atheroma of the Aorta in ApoE^−/−^ Mice

**Figure 6:**
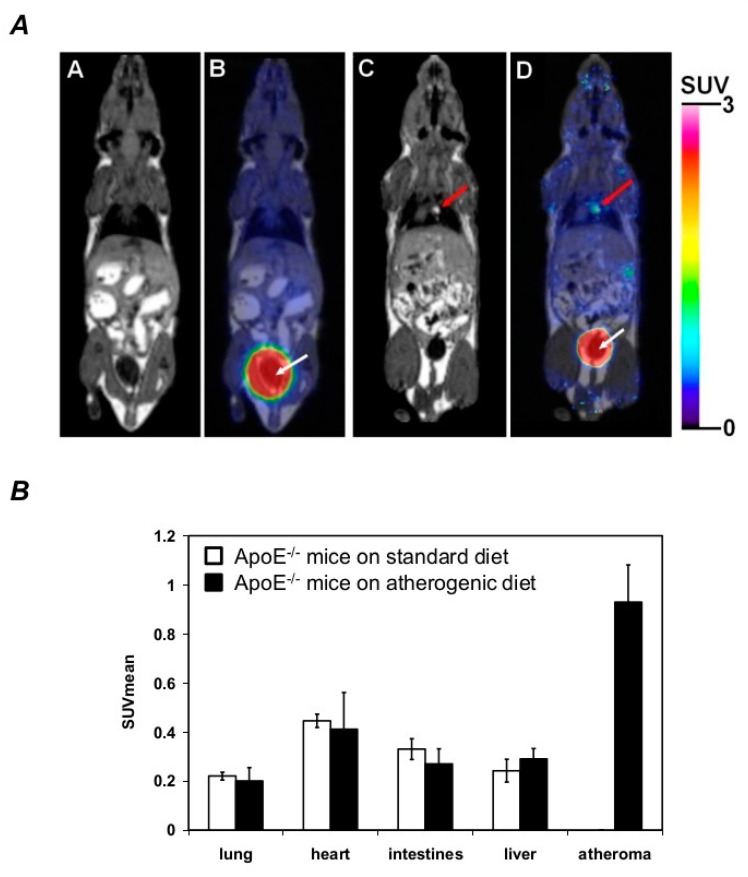
DFC accumulates within the atheromatous plaque of aorta in ApoE^−/−^ mice. ApoE^−/−^ mice were maintained on an atherogenic diet (*n* = 5) for 8 weeks to develop atheromatous plaques within the aorta. For control ApoE^−/−^ mice were on a standard diet (*n* = 5) for 8 weeks. ^68^Ga-DFC was administered for both ApoE^−/−^ mice kept on an atherogenic diet and ApoE^−/−^ mice on a standard diet via the lateral tail vein. (**A**) After administration of ^68^Ga-DFC, representative T1-weighted MRI and coregistered PET-MRI images of ApoE^−/−^ mice on an atherogenic diet are shown in Panel C and D, respectively. Scale bar: 1 cm. (**A**) Representative T1-weighted MRI and coregistered PET-MRI images of ApoE^−/−^ mice on a standard diet treated with ^68^Ga-DFC are shown in Panels A and B, respectively. Red arrows indicate atheroma of aortic arch; white arrows indicate bladder with urine. (**B**) Quantitative analysis of PET/MRI images of heart, lung, intestines, liver and atheromas at 30 min of intravenous injection of ^68^Ga-DFC.

To demonstrate the localization of DFC, we employed PET/MRI imaging using ^68^Ga-DFC. We kept ApoE^−/−^ mice on an atherogenic diet for eight weeks to develop atheromatous plaques within the aorta. For control, ApoE^−/−^ mice were maintained on a standard diet. At the age of eight weeks, ^68^Ga-DFC was administered via the lateral tail vein for both groups. Qualitative analysis of T1-weighted MRI images visualized atheromatous plaque within the aortic arch of ApoE^−/−^ mice on an atherogenic diet (Figure 6A, panel C–D). On the contrary, atheromatous plaque formation did not occur in the aorta of ApoE^−/−^ mice on a standard diet (Figure 6A, panel A,B). Following the analysis of the decay-corrected PET images, specific ^68^Ga-DFC accumulation was found in the atheromas of ApoE^−/−^ mice on an atherogenic diet (Figure 6A, panel D). In contrast, ^68^Ga-DFC was not detected in animals on a standard diet (Figure 6A, panel B). The quantitative analysis of PET images confirmed the accumulation of ^68^Ga-DFC within the atheromas of ApoE^−/−^ mice on an atherogenic diet (SUVmean: 0.93 ± 0.15 and SUVmax: 1.38 ± 0.21, Figure 6B). As we compared ApoE^−/−^ mice on an atherogenic diet to ApoE^−/−^ mice on a standard diet no significant differences were found in the amount of ^68^Ga-DFC associated with lung, heart, intestines and liver (Figure 6B).

## 3. Discussion

Lipid peroxidation and the subsequent vascular inflammation play a crucial role in the fate of the atherogenesis [1,17,20]. Since there is no free radical injury without transition metals, and iron chelators might mitigate plaque formations, iron has gained a lot of interest in atherosclerosis research. Our team is one of those groups who studied the origin of redox-active iron in pathological conditions. We raised first in the literature that heme, a natural hydrophobic iron complex, could be the source of the redox-active labile iron pool. During extravasation of red blood cells, hemolysis occurs and oxidation of liberated Hb leads to the formation of metHb and ferrylHb followed by heme release that catalyzes the oxidation of atherosclerotic plaque materials [40,41]. Moreover, in intravascular hemolysis, oxidation of Hb results in translocation of heme moieties to LDL particles generating oxLDL (48). In this process, heme degradation and subsequent iron release occur that is implicated in vascular endothelial cell damage [3,37,65,66]. The relationship between nonprotein-bound iron and the risk of cardiovascular disease development and progression is well documented [10,33,67]. This supports the hypothesis that the chelation of redox-active iron with siderophores may lower the development of atherosclerosis and decrease cardiovascular disease morbidity and mortality by attenuating iron-mediated redox reactions [68,69].

Evidence suggests that iron chelation has a therapeutic potential to attenuate atherosclerosis. Deferiprone, an iron chelator used to treat iron overload in thalassemia major protects endothelial cells against oxLDL-induced cytotoxicity and reduces cholesterol levels in a hypercholesterolemic rabbit model, however, no differences have been detected between deferiprone-treated and control animals in the surface area of plaque involvement by planimetry [70]. Others have shown that desferrioxamine (DFO), a siderophore from *Streptomyces pilosus*, inhibits atherosclerotic lesion development in cholesterol-fed rabbits [71] as well as in ApoE knockout mice [48]. However, DFO has a positively charged free amino group (pK = 10.84) [72] at physiologic pH resulting in possibly poor penetration across biological membranes or into the lipid-rich environment of atherosclerotic plaques. Furthermore, DFO treatment has severe side effects such as pain and infusion reactions after intravenous administration [73] or even hepatotoxicity [74]. In contrast to DFO, desferricoprogen (DFC), a siderophore from *Neurospora crassa*, has a more hydrophobic character which enables its incorporation into LDL particles [52] and possibly its transmembrane penetration. These aforementioned characteristics of DFC led us to hypothesize that DFC might penetrate the lipid-rich area of atheromas and possibly counteracts the toxic effects of free heme, a natural hydrophobic iron complex, as well as ameliorates oxidative stress. As demonstrated by small animal PET/MRI, we found that DFC could reach the atheromatous lesions of the aorta in ApoE^−/−^ mice. An interesting aspect of this finding is that DFC, possibly due to its hydrophobic nature at physiologic pH, accumulates in atheromas as early as 30 min after the ^68^Ga-DFC administration suggesting its rapid translocation into the lipid-rich environment of plaques. Besides, our results suggest that ^68^Ga-DFC possesses desired pharmacokinetic properties (fast blood clearance, urinary excretion, high uptake in atheromas). We postulate that ^68^Ga-DFC might represent a promising candidate diagnostic tracer for PET/CT imaging of the atherosclerotic lipid burden and might be a tool for the diagnosis of plaque with PET. Hence, it would be interesting for future studies to compare ^68^Ga-DFC as a potential diagnostic tracer with other tracers such as ^18^F-Fluorodeoxyglucose [75], Translocator Protein Ligands [76], or 68Ga-DOTATATE [77].

To provide a more comprehensive understanding of the possible protective role of DFC in atherosclerosis, our studies focused first on atheroma development in ApoE^−/−^ mice. As demonstrated, we found that DFC attenuated atherogenic diet-induced atherosclerotic plaque formation in ApoE^−/−^ mice. Lipid accumulation in the vessel wall and its extensive peroxidation [4,78], as well as expression of proteins involved in fibrous cap formation (elastin, collagen) [79] and monocyte adhesion to the endothelium [80], are pivotal determinants of plaque formation. To gain possible mechanistic insight into this phenomenon, we analyzed early (lipid accumulation and peroxidation, adhesion molecule expression on endothelial cells) as well as late factors (fibrous cap formation) of plaque development in DFC-treated mice on an atherogenic diet. Similar to DFO in rabbits [71] and mice [48], DFC did not affect serum cholesterol levels in ApoE^−/−^ mice, however, significantly inhibited lipid accumulation in the vessel wall. The intima of vessels directly interacts with proatherogenic substances both in the bloodstream and in the subendothelial space inducing adhesion molecule expression on endothelial cells facilitating the transendothelial migration and differentiation of monocytes into inflammatory macrophages in the subendothelial area that contributes to the development of atherosclerosis. Our findings showed that DFC inhibited VCAM-1 expression in ApoE^−/−^ mice. This is in good agreement with a previous study which has demonstrated that DFO also prevents VCAM-1 activation in ApoE^−/−^ mice [48]. The present study shows that DFC effectively attenuates both early and late events of atherosclerosis.

Solid evidence suggests that lipid peroxidation is a crucial step of atherosclerosis [3,4]. We have previously described that DFC inhibits heme-catalyzed LDL oxidation in vitro [52]. Importantly, the composition of plaque lipids is more complex containing different lipid forms [81]. Here, we observed that DFC effectively attenuated heme or Hb-catalyzed plaque lipid peroxidation. In contrast, iron-saturated FC failed to inhibit plaque lipid peroxidation. This suggests that the antioxidant activity of DFC is dependent on its iron-binding ability. In line with these in vitro results, we demonstrated that DFC significantly lowered 4-HNE, an endproduct lipid peroxidation, content of the aortas in mice. 4-HNE is certainly involved in the pathogenesis of atherosclerosis [82] by triggering inflammatory [83] or even cell death signalings [84]. Elevated oxLDL plasma level is an independent risk factor for atherosclerosis [85,86]. High plasma and plaque oxLDL levels correlate with the vulnerability to rupture of atherosclerotic plaques [87] as well as with the severity of acute coronary syndromes [88]. The increase of plasma oxLDL level is also observed in ApoE^−/−^ mice during the progression of atherosclerosis [89]. Here, we observed that DFC treatment significantly lowered oxLDL plasma levels in mice compared to the controls. Statins are generally being used as lipid-lowering medications to reduce cardiovascular disease. Simvastatin [90], as well as rosuvastatin and atorvastatin [91], are reported to lower circulating ox-LDL levels. Similar results were found by Ndrepepa et al. who demonstrated that patients who received statins had lower circulating ox-LDL levels and less severe coronary artery disease compared to the controls who did not receive statins [92]. These are in good agreement with our results in mice that support our hypothesis that the protective effect of DFC in mice, at least in part, can be attributed to the circulating oxLDL-lowering effect of DFC.

OxLDL is known to trigger oxidative stress in sublethal concentration and induces HO-1 expression, a well-established marker of oxidative injury, or even activates cell death signaling [58]. In our previous work, we have shown that DFC effectively inhibits oxLDL-induced oxidative injury and cell death in endothelial cells [52]. In the present study, we corroborated our previous findings on endothelial cells. Interestingly, a significant difference was observed between endothelial cells and macrophages regarding oxLDL-induced cell death that is macrophages were more susceptible to oxLDL resulting in increased cell death compared to endothelial cells, however, DFC also effectively counteracted the more robust cell death in macrophages.

Macrophages predominantly transport oxLDL through the scavenger receptor CD36 [93], which is implicated in atherogenesis [61]. Besides, oxLDL upregulates the expression of its receptor CD36 in macrophages [62] and triggers inflammatory signaling through CD36 [94] generating atherosclerosis [95]. TNF-α, a well-known proinflammatory cytokine, is actively involved in atherosclerosis [64]. Inhibition of TNF-α reduces atherosclerosis in ApoE^−/−^ mice [64]. TNF-α also enhances macrophage foam cell formation possibly by inhibiting intracellular lipid catabolism [96]. The present study corroborated our previous findings that oxLDL induces CD36 expression and robust TNF-α release in macrophages. Importantly, our results presented here showed that DFC inhibited CD36 and TNF-α expression in macrophages in response to oxLDL accompanied by the reduction of foam cell formation. Therefore, we postulate that inhibition of foam cell formation by DFC, at least in part, is attributed to its suppressive effect on CD36 and TNF-α expression.

Endothelial cells express a wide variety of cellular adhesion molecules such as VCAM-1, ICAM-1 and E-selectin in response to TNF-α resulting in impaired endothelial integrity [55,56]. These adhesion molecules recruit circulating monocytes and facilitate their trans-endothelial migration triggering oxLDL-driven foam cell formation creating a proinflammatory scenario in plaques [15,97]. Here, we showed that DFC preserved endothelial integrity and counteracted with TNF-α to impair endothelial integrity and inhibited monocyte adhesion to the endothelial cells in vitro. DFC also lowered VCAM-1 expression in the aortas of the ApoE^−/−^ mice. It supports previous findings that have shown that inhibition of adhesion molecule expression attenuates early atherosclerosis both in vitro [98] and animal models [99]. Based on these results, it is reasonable to assume that DFC inhibits atherosclerosis, at least in part, via blunting adhesion molecule expression.

In this study, we applied an established and highly reproducible in vivo (ApoE^−/−^ mice on a high-fat diet) model of atherosclerosis to investigate the potential antiatherosclerotic effect of DFC, a siderophore from *Neurospora crassa*. Taken together, our results provide evidence that DFC inhibits atherosclerotic plaque formation by lowering the oxLDL plasma level, inhibiting lipid accumulation as well as atherosclerosis-driven increased collagen and elastin expression in the vessel wall and preventing adhesion molecule expression. These findings were corroborated by cell culture, *ex vivo,* and test-tube experiments which also demonstrated the protective effect of DFC by inhibiting LDL- and plaque lipid oxidation as well as oxLDL-induced foam cell formation and TNF-α induction. Besides, DFC preserved endothelial integrity and prevented adhesion molecules as well as CD36 expression on endothelial cells which blunted monocyte adhesion to the cells. Another interesting finding of our work that ^68^Ga-DFC accumulated in aortic lesions of ApoE^−/−^ mice on a high-fat diet and showed fast blood clearance, urinary excretion and high uptake in atheromas that might be a promising candidate diagnostic tracer for PET/CT imaging of the atherosclerotic lipid burden in future studies. DFC may expand our armamentarium to prevent cardiovascular and atherosclerotic diseases.

## 4. Materials and Methods

### 4.1. Materials

All reagents were purchased from Sigma-Aldrich (Darmstadt, Germany) with ACS grade or higher purity, except when otherwise specified below.

### 4.2. Study Approval

Collection of carotid artery plaques from patients who underwent carotid endarterectomy was approved by the Scientific and Research Ethics Committee of the Scientific Council of Health of the Hungarian Government under the registration number of DE OEC RKEB/IKEB 3712-2012 (approved on 25 September 2012). Written consent was received from the participants according to the Declaration of Helsinki. Specimens were classified according to the AHA guidelines. Healthy segments and type IV (atheromatous) lesions were selected for further investigation.

### 4.3. Mice

All the animal experiments were approved by the guidelines from Directive 2010/63/EU of the European Parliament on the protection of animals used for scientific purposes and by the Scientific and Research Ethics Committee of the Scientific Council of Health of the Hungarian Government under the registration number of DE MÁB/157-5/2010 (approved on 30 December 2010) and reported by the ARRIVE guidelines. Apolipoprotein E-deficient mice (ApoE^−/−^) on the C57BL6 background were purchased from Jackson Laboratories (Bar Harbor, ME, USA) and maintained under specific pathogen-free conditions at the University of Debrecen. Atherosclerotic plaque formation was induced by ad libitum administration of an atherogenic diet containing 5% fat and 1.2% cholesterol (ssniff Spezialdiäten GmbH, Soest, Germany) at the age of 8 weeks. Mice were divided into two groups, one receiving intraperitoneal DFC (160 mg/kg) or physiological saline every second day. Aortas from both groups were collected after 8 weeks of treatment. Mice were euthanized by controllable administering slow-fill CO_2_ asphyxiation.

### 4.4. Coprogen Production, Purification and Deferration

*Neurospora crassa* cultivated in defined low-iron minimal media containing 5g/L aspartate, 1 g/L K_2_HPO_4_ × 3H_2_O, 1 g/L MgSO_4_ × 7H_2_O, 0.5 g/L CaCl_2_ × 2H_2_O, 0.02 g/L ZnSO_4_ × 7H_2_O, 10 μg/L biotin and 20 g/L glucose; pH set to 7.0 with NaOH [100]. Ferricoprogen purification was performed using Amberlite XAD-2, Kieselgel 60, and Bio-Gel P-2 liquid chromatographies and preparative high-performance liquid chromatography (HPLC) on a Supercosil-Si matrix. The purity of ferricoprogen was checked with HPLC then pure ferricoprogen was deferrated using methanolic 8-hydroxyquinoline, lyophilized and stored at −70 °C.

### 4.5. Quantification of Aortic Lesions in ApoE^–/–^ Mice

Aortas were dissected from the aortic arch to the iliac bifurcation. After that, the fatty tissue was carefully removed under a stereomicroscope (M125; Leica Biosystems Nussloch GmbH). Atherosclerotic lesions were identified by en face Oil Red O (ORO; Sigma-Aldrich, Lot. O0625;) staining for which 0.07 g ORO powder was dissolved in 25 mL methanol and mixed for 10 min on a magnetic stirrer. Finally, 10 mL of 1 mol/L NaOH was added to the ORO solution followed by filtration. Images were taken with Nikon D3200 camera (Nikon Corp., Japan) and the entire aorta plaque area was analyzed using ImageJ 1.49 software.

### 4.6. Lipids Extraction from the Human Carotid Arteries

The extraction of lipids was performed as previously described [101].

### 4.7. Cell Culture

Human Umbilical Vein Endothelial Cells (HUVEC, Debrecen, Hungary) were isolated from umbilical veins by exposure to dispase and cultured in CM199 medium containing 10% FBS, antibiotics, L-glutamine, sodium pyruvate and endothelial cell growth factor as described previously [101]. HUVEC were used at passage 3 after one-day postconfluence. RAW264.7 cells were cultured (American Type Culture Collection (ATCC), Manassas, VA, USA) in DMEM supplemented with 10% FBS, antibiotics, L-glutamine and sodium pyruvate according to the manufacturer’s guide.

### 4.8. Immunohistochemistry

Immunohistochemistry was performed on formalin-fixed, tissue freezing medium-embedded (Leica Biosystems Nussloch GmbH) cryosections (6 μm) of mouse aortic roots. Standard hematoxylin (Sigma-Aldrich, Lot. GHS280) and eosin (Sigma-Aldrich, Lot. HT110116), elastic (Sigma-Aldrich, Lot. HT25A), ORO and Masson trichrome (Sigma-Aldrich, Lot. HT15) stainings were performed.

### 4.9. Measurement of the Plasma Cholesterol and oxLDL Levels

Plasma oxLDL levels were measured by ELISA (EA527Hu; Wuhan USCN Business Co., Ltd.; Wuhan, Hubei, China). Plasma HDL-C, LDL-C, cholesterol and triglycerides levels of mice were measured employing Hitachi cobas 8000 instrument (Roche).

### 4.10. Immunofluorescent Staining

Aortic root cryosections of mice were fixed in 3.7% formaldehyde for 2 h, then were permeabilized with 0.3% Triton X-100 for 30 min. Samples were then blocked with 5% goat serum (Jackson ImmunoResearch Europe Ltd., Suffolk, UK) for 30 min at room temperature. For Vascular Cell Adhesion Molecule-1 (VCAM-1) and 4-Hydroxynonenal (4-HNE) stainings, samples were incubated with rabbit polyclonal VCAM-1 (H-276, Santa Cruz Biotechnology, Inc. Dallas, U.S.A, 1:250 dilution) or rabbit polyclonal 4-HNE (ab46545, Abcam PLC. Cambridge, UK 1:200 dilution,) antibodies for 1 h at room temperature followed by incubation with Alexa Flour 568 F(ab’)2 fragment of goat antirabbit IgG (H+L, Cat no. A11004, Thermo Fisher Scientific Inc., Waltham, MA U.S.A.; 1:500 dilution) secondary antibody for 1 h at room temperature. F-actin was stained with FITC-conjugated phalloidin, while DNA (nuclei) was stained with Hoechst 33,258 (50 ng/mL, H6024). Images were taken with a fluorescent microscope at a magnification of 400× (DM2500, Leica Microsystems GmbH, Wetzlar, Germany). The brightness of all immunofluorescent staining was equally adjusted.

### 4.11. Hemoglobin Preparation

Briefly, Hb was isolated from freshly drawn blood of the healthy volunteers using ion-exchange chromatography on a DEAE Sepharose CL-6B column (DCL6B100-500Ml, Sigma-Aldrich, St. Louis, MO) [101]. Purified Hb was dialyzed against physiological saline (3 times for 3 h at 4 °C) and concentrated using Amicon Ultra centrifugal filter tubes (15 kDa MWCO, Millipore Corp., Billerica, MA, USA). Aliquots were snap-frozen in liquid nitrogen and stored at −70 °C. The purity of each Hb preparations was verified using SDS-PAGE followed by staining with ProteoSilver Plus Silver Staining Kit (PROTSIL1-1KT, Sigma-Aldrich, St. Louis, MS, USA). The purity of Hb preparations was above 99.9%. Hb concentration was calculated as described by Winterbourn [102].

### 4.12. Preparation and Oxidation of LDL

LDL was isolated from the plasma of EDTA-anticoagulated venous blood of healthy volunteers by gradient ultracentrifugation (Beckman Coulter, Inc., Brea, CA, USA) [3]. The density of plasma was adjusted to 1.3 g/mL with KBr. Two gradient layers were made in a Quick-Seal ultracentrifuge tube by layering physiological saline on the plasma. Ultracentrifugation was performed at 302,000 × g for 2 h at 4 °C (VTi 50.2 rotor). Protein concentrations of the LDL samples were determined by the Pierce BCA protein assay kit (Pierce Biotechnology, Rockford, IL, USA). LDL oxidation (200 µg/mL) was carried out by the addition of heme (5 µmol/L) and H_2_O_2_ (75 µmol/L) in the presence or absence of DFC (50 µmol/L) or iron-saturated ferricoprogen (FC, 50 µmol/L) at 37 °C for 2 h.

### 4.13. Measurement of the Lipid Peroxidation Products

Conjugated diene formation was being continuously monitored for 2 h at 234 nm. Delta OD 234 nm was calculated by subtracting optical density measured at a 0-time point from optical density measured for 2 h. The formation of lipid hydroperoxides (LOOH) in the LDL samples was measured by Simon-Wolf reagent [3]. For measuring the sample thiobarbituric-acid-reactive substances (TBARS), 50 μL of a 200 µg protein/mL LDL was mixed with 100 μL of thiobarbituric acid reagent (0.375 g 2-thiobarbituric acid, 2.08 mL HCl, 15 mL 10% trichloroacetic acid to a final volume of 100 mL). Following the above steps, the samples were heated at 90 °C for 15 min followed by extraction with 200 μL n-butanol. The upper phase was measured spectrophotometrically at 532 nm. The amount of TBARS was calculated using a molar extinction coefficient of 1.56 × 10^5^ M^−1^cm^−1^ and is expressed as nmol TBARS/mg LDL or nmol TBARS/mg PL.

### 4.14. Isolation and Oxidation of Human Plaque Lipids

Lipids from human atherosclerotic lesions were extracted as described previously [103]. Lipids derived from the atheromatous lesion (1 mg/mL) were incubated with heme (5 µmol/L) or Hb (5 µmol/L) in the presence or absence of DFC (50 µmol/L) or FC (50 µmol/L) at 37 °C for 16 h. Lipid peroxidation was assessed by measuring LOOH and TBARS content.

### 4.15. Western Blot

HUVEC were cultured in 6-well plates. Confluent cells were then incubated with or without DFC (50 µmol/L) for 16 h in CM199 medium containing 5% FBS then incubated with or without tumor necrosis factor-α (TNF-α, 1 ng/mL, X01394, R&D Systems, Inc. Minneapolis, MN, USA) for 8 h. Cells were then solubilized in 10 mmol/L Tris-HCl, containing 5 mmol/L EDTA, 150 mmol/L NaCl (pH 7.4), 10% Triton X-100, 5% Nonidet P-40, and protease inhibitors (Complete Mini, F. Hoffmann-La Roche Ltd., Basel, Switzerland). In another set of experiments, cells were pretreated with DFC as described above then exposed to native LDL (50 µg/mL) or oxLDL (50 µg/mL) for 6 h. Proteins (15 µg) were applied to 12.5% SDS-PAGE gels or 6% native PAGE gel. After electrophoresis, proteins transferred to a 0.22 µm nitrocellulose membrane (Amersham Biosciences Corp., Piscataway, NJ, USA). VCAM-1, ICAM-1, E-selectin or HO-1 expressions were examined using a rabbit antihuman VCAM-1 (1:200 dilution, sc-8304, Santa Cruz Biotechnology Inc., Dallas, Texas, USA), mouse antihuman ICAM-1 (1:500 dilution, ab2213, Abcam PLC. Cambridge, UK), rabbit antihuman E-selectin (1:500 dilution, sc-14011, Santa Cruz Biotechnology Inc., Dallas, TX, USA) or rabbit antihuman HO-1 (1:2500 dilution, Cat No. 374087, Calbiochem, MerckKGaA, Darmstadt, Germany) antibodies. To ascertain equal protein loading, membranes were stripped and reprobed with mouse antihuman glyceraldehyde-3-phosphate dehydrogenase (GAPDH) antibody (1:1000 dilution, N13300-221, Novus Biologicals, LLC, Littleton, CO, USA). Protein bands were visualized by horseradish peroxidase-conjugated donkey antirabbit or goat antimouse IgG secondary antibodies. Antigen-antibody complexes were detected by a horseradish peroxidase chemiluminescence system according to the manufacturer’s instructions (Amersham Biosciences Corp., Piscataway, NJ, USA). Quantification was performed using video densitometry (AlphaDigiDoc RT, Alpha Innotech Corp., San Leandro, CA, USA).

### 4.16. Endothelial Cell Monolayer Integrity Assay

To measure endothelial monolayer integrity, we used Electric Cell-substrate Impedance Sensing (ECIS) method. Confluent HUVEC were pretreated in the presence or absence of DFC (50 µmol/L) for 16 h in medium containing 5% FBS. Then, cells were incubated in the presence or absence of TNF-α (1 ng/mL) for 6 h. The complex impedance spectrum was monitored with an ECIS Zθ instrument (Applied BioPhysics Inc., Troy, NY, USA) over 3 h. Intracellular gap formation was calculated based on the difference between monolayer resistance at 4000 Hz at 0-time points and 3 h.

### 4.17. Monocyte Adhesion Assay

Confluent HUVEC were incubated with or without DFC (50 µmol/L) for 16 h in CM199 medium containing 5% FBS. Then, cells were incubated with or without TNF-α (1 ng/mL) for 6 h. Peripheral blood mononuclear cells (PBMCs) were prepared by using Histopaque-1077 (10771, Sigma-Aldrich, St. Louis, MO). Following that, CD14-positive cells were separated using a magnetic isolation procedure (MACS CD14 microbeads, Miltenyi Biotec GmbH, Bergisch Gladbach, Germany). As a next step, monocytes were suspended in Hank’s Balanced Salt Solution (HBSS+) containing Ca^2+^ and Mg^2^ at a density of 5 × 10^5^ cells/mL and stained with calcein-AM (C17783, Sigma-Aldrich, St. Louis, MO) in a final concentration of 5 µmol/L for 30 min at 37 °C. Calcein-labeled monocytes were then coincubated with HUVECs in HBSS+ for 30 min at 37 °C. Nonadherent cells were washed away with HBSS+. Cells were then fixed with 4% formalin for 15 min and F-actin was stained with TRITC-conjugated phalloidin (25 ng/mL) while DNA (nuclei) was stained with Hoechst (0.5 ng/mL, H6024). Images were taken with a fluorescent microscope at a magnification of 400× (DM2500, Leica Microsystems GmbH, Wetzlar, Germany).

### 4.18. Viability Assay

HUVEC and RAW264.7 cells were cultured in 96-well plates. Confluent cells were then cultured in the presence or absence of DFC (50 µmol/L) for 16 h in CM199 or DMEM containing 5% FBS. Next, cells were treated with native LDL (75 µg/mL) or oxLDL (75 µg/mL) in HBSS+ for 6 h. Cell viability was assessed by MTT assay as described previously [104].

### 4.19. Quantitative Real-Time Polymerase Chain Reaction (qRT-PCR)

RAW 264.7 cells were grown in 6-well plates and pretreated with DFC (50 µmol/L) for 16 h. Cells were then exposed to native LDL (50 µg/mL) or oxLDL (50 µg/mL) for 6 h, washed twice with phosphate-buffered saline pH 7.4 (PBS), and total RNA was isolated using RNAzol STAT-60 following the manufacturer’s instructions (Cat. No. Tl-4120, TEL-TEST Inc., Friendswood, TX, USA). RNA concentration was measured with the NanoDrop^TM^ 2000c spectrophotometer (Cat. No.S06497c, Thermo Scientific Inc. Waltham, MA, U.S.A.). Next, cDNA synthesis was performed using a high capacity cDNA kit (Cat. No. 43-688-13, Applied Biosystems, Foster City, CA). HO-1, CD36, TNF-α and GAPDH mRNA levels were estimated with quantitative real-time PCR. Finally, we performed TaqMan quantitative PCR with the Bio-rad CFX96 (Bio-Rad Laboratories, Inc. Hercules, CA, USA) detection system. The results were expressed as mRNA expression normalized to GAPDH. Specific primers were purchased from Thermo Fisher Scientific Inc., while TaqMan Universal PCR Master Mix was purchased from Applied Biosystems (Cat. No. 4269510, Applied Biosystems, Foster City, CA, USA).

### 4.20. Macrophages Lipid Uptake Measurement

RAW 264.7 cells were cultured in 10% FBS containing DMEM in 12-well plates. Upon macrophages reaching 70% of confluence, cells were preincubated with DFC (50 µmol/L) or FC (50 µmol/L) for 16 h in HBSS+. After that, RAW 264.7 cells were exposed to LDL (100 µg/mL)-heme (5 µmol/L) solution with or without DFC (50 µmol/L), FC (50 µmol/L) for 24 h. To reveal the lipid droplets in macrophages, ORO staining was performed as described above.

### 4.21. Small Animal PET/MRI

The gallium generator was obtained from Eckert and Ziegler. It was eluted with 5 mL 0.1 mol/L HCl (ultrapure, Roth GmbH), resulting in 120–140 MBq total activity. The ^68^Ga content of the eluate was trapped on a Strata SCX cartridge (Phenomenex, 100 mg bed weight), and eluted with 400 µL ethanol (cc.HCl 9:1). The ethanolic gallium solution was evaporated to dryness by the aid of nitrogen gas (500 mL/min) in a Teflon capillary (length: 2 m, I.D.: 0.8 mm) heated to 100 °C. The activity was washed out from the capillary with 100 µL DFC solution, prepared by mixing 10 µL 1 mg/mL DFC solution and 90 µL pH = 4 ammonium acetate buffer (3.5 mol/L). The solution was collected in an Eppendorf vial, and heated to 95 °C for 5 min. The reaction mixture was transferred to a Strata-X C18 cartridge. It was washed with 1 mL ammonium acetate buffer, and the product eluted with 400 µL ethanol. The ethanol was evaporated to dryness in a heated Teflon capillary for one minute. The product was redissolved in 100 µL PBS. Radiochemical purity was determined by HPLC using a Waters Acquity CSH C18 column and a linear gradient from 0.1% phosphoric acid to acetonitrile in 5 min. RCY 62.2 ± 4.3; radiochemical purity was 100%.

### 4.22. Small Animal PET/MRI Imaging Using ^68^Ga-DFC

ApoE^−/−^ mice were maintained on a standard diet (*n* = 5) or on an atherogenic diet (*n* = 5) for 8 weeks to develop atheromatous plaques within the aorta. ^68^Ga-DFC was administered via the lateral tail vein in 0.15 mL volume after mice were anesthetized with 3% isoflurane with a dedicated small animal anesthesia device. Entire body PET scans (10 min static PET scans) were acquired using the preclinical nanoScan PET/MRI system (Mediso Ltd., Budapest, Hungary) 30 min after the ^68^Ga-DFC administration. To prevent the motion of the animals, mice were fixed to a mouse chamber (MultiCell Imaging Chamber, Mediso Ltd., Budapest, Hungary) and positioned in the center of the field of view (FOV). For the determination of the anatomical localization of the organs and tissues, T1-weighted MRI scans were performed (3D GRE EXT multi-FOV; TR/TE 15/2 ms; FOV 40 mm; NEX 2). PET volumes were reconstructed using a three-dimensional Ordered Subsets Expectation Maximization (3D-OSEM) algorithm (Tera-Tomo, Mediso Ltd., Hungary). PET and MRI images were automatically coregistered by the PET/MRI instrument’s acquisition software (Nucline). Reconstructed, reoriented and coregistered images were further analyzed with InterView™ FUSION (Mediso Ltd., Hungary) image analysis software.

### 4.23. PET Data Analysis

The radiotracer uptake was expressed in terms of standardized uptake values (SUVs). Ellipsoidal 3-dimensional Volumes of Interest (VOI) were manually drawn around the edge of the tissue or organ activity by visual inspection using InterView™ FUSION multimodal visualization and evaluation software (Mediso Ltd., Hungary). The standardized uptake value (SUV) was calculated as follows: SUV = [VOI activity (Bq/mL)]/[injected activity (Bq)/animal weight (g)], assuming a density of 1 g/mL.

### 4.24. Experimental Units

“N” represents the number of tissue samples used in each group. The “n” denoting the number of replications of the independent results.

### 4.25. Statistics

Data were analyzed by GraphPad Prism 5.02 software (GraphPad Software Inc., San Diego, CA, USA). All statistics data are expressed as mean ± SEM. If data groups passed the normality test and equal variance test, we performed Student’s t-test or one-way ANOVA followed by Bonferroni post hoc tests as indicated in figure legends. *p* < 0.05 was considered significant.
